# Current Insights Regarding the Role of Farm Animals in the Spread of Antimicrobial Resistance from a One Health Perspective

**DOI:** 10.3390/vetsci9090480

**Published:** 2022-09-05

**Authors:** Mohamed Rhouma, Leila Soufi, Schlasiva Cenatus, Marie Archambault, Patrick Butaye

**Affiliations:** 1 Department of Pathology and Microbiology, Faculty of Veterinary Medicine, Université de Montréal, Saint-Hyacinthe, QC J2S 2M2, Canada; 2 Groupe de Recherche et d’Enseignement en Salubrité Alimentaire (GRESA), Faculty of Veterinary Medicine, Université de Montréal, Saint-Hyacinthe, QC J2S 2M2, Canada; 3 Swine and Poultry Infectious Diseases Research Center, Faculty of Veterinary Medicine, Université de Montréal, Saint-Hyacinthe, QC J2S 2M2, Canada; 4 Department of Microbiology, Faculty of Life Sciences and Technology, Berlin University of Applied Sciences, Seestrasse 64, 13347 Berlin, Germany; 5 Laboratory of Biotechnology and Bio-Geo Resources Valorization (BVBGR)-LR11ES31, Higher Institute for Biotechnology, University of Manouba, Biotechpole Sidi Thabet, Ariana 2020, Tunisia; 6 Department of Pathobiology, Pharmacology and Zoological Medicine, Faculty of Veterinary Medicine, Ghent University, Salisburylaan 133, B9820 Merelbeke, Belgium

**Keywords:** antimicrobials, antimicrobial resistance, antimicrobials use, farm animals, One Health

## Abstract

**Simple Summary:**

Antimicrobial resistance is of critical concern for both human and veterinary medicine worldwide. Many bacterial infections are currently very difficult to treat due to the presence of several mechanisms involved in bacterial resistance to marketed antimicrobials. Preserving the effectiveness of currently available antimicrobials and reducing the burden of infections caused by resistant bacteria is a priority for competent authorities operating in both sectors. It has been perceived that the livestock sector is a primary contributor to the spread of bacterial resistance in both humans and the environment. This review examined the recent scientific literature on this topic in order to explore the relationship between antimicrobials use in farm animals and the selection of bacterial resistance in this sector and its subsequent dissemination to humans. Recent data indicated that the global biomass-adjusted amount of antimicrobials consumed by farm animals was slightly higher than the amounts used in humans, while the reel contribution of farm animals in the spread of AMR to humans is probably very low compared to initial estimations. However, this review highlights the importance of the close collaboration between veterinary and human medicine, as part of the ‘One Health’ approach, to preserve the longevity of antimicrobials.

**Abstract:**

Antimicrobial resistance (AMR) represents a global threat to both human and animal health and has received increasing attention over the years from different stakeholders. Certain AMR bacteria circulate between humans, animals, and the environment, while AMR genes can be found in all ecosystems. The aim of the present review was to provide an overview of antimicrobial use in food-producing animals and to document the current status of the role of farm animals in the spread of AMR to humans. The available body of scientific evidence supported the notion that restricted use of antimicrobials in farm animals was effective in reducing AMR in livestock and, in some cases, in humans. However, most recent studies have reported that livestock have little contribution to the acquisition of AMR bacteria and/or AMR genes by humans. Overall, strategies applied on farms that target the reduction of all antimicrobials are recommended, as these are apparently associated with notable reduction in AMR (avoiding co-resistance between antimicrobials). The interconnection between human and animal health as well as the environment requires the acceleration of the implementation of the ‘One Health’ approach to effectively fight AMR while preserving the effectiveness of antimicrobials.

## 1. Introduction

The introduction of antimicrobials for clinical use is certainly the greatest medical breakthrough of the 20th century, being responsible for the treatment of serious bacterial diseases, as well as for major advances in surgery (e.g., organ transplants and open-heart surgery) [1]. However, the invaluable benefits of antimicrobials over the past few decades, in both humans and animals, are being increasingly threatened by the selection and spread of antimicrobial resistance (AMR), with some infections now effectively becoming untreatable [2]. Some reports had estimated that about 26% of bacterial infections in humans are currently resistant to first-line antimicrobials [3]. In addition, several recent alarming studies have highlighted the global burden of the current rather silent AMR pandemic. Murray et al. (2022) estimated a median of 1.27 million deaths in 2019 directly attributable to AMR [4], a value that is roughly the same as HIV deaths (680,000) and malaria deaths (627,000) combined on a global scale. This number is expected to increase to 10 million in 2050 if no action is undertaken [2,5]. On the other hand, no new class of antimicrobial has been discovered since daptomycin and linezolid in the 1980s which can alleviate the burden of AMR, and only the optimization of the chemical structure or combination of already known compounds have recently been commercialized [6]. Hope remains for antimicrobial peptides (AMPs), which have attracted great interest in recent years due to their promising potential; it is understood that they could be used as alternative or complement approaches for the treatment of bacterial infections [7].

Antimicrobials have been used, since the 1950s, therapeutically, prophylactically, and even for growth promotion in farm animals to maintain animal health and increase overall productivity [8,9]. The misuse of antimicrobials in human health care and in livestock is believed to be the major driver of AMR [10]. It is noteworthy that despite the current critical worldwide situation related to the COVID-19 pandemic, and the unprecedented efforts and budgets dedicated to managing this crisis, AMR was a priority of the health agenda of both the June 2021 G7 and the September 2021 G20 meetings [11,12], testifying to the awareness of policymakers regarding the impact of the silent pandemic of AMR on the global healthcare system. 

Several studies have reported that the application of different strategies aimed at reducing the use of antimicrobials in veterinary medicine (for example, the use of colistin in farm animals) have positively contributed to a decrease in the prevalence of colistin-resistant *E. coli* and the mobile colistin resistance (*mcr)-1*-positive *E. coli* in animals, humans, and the environment [13,14]. In the Netherlands, an overall decrease of the use of antimicrobials by approximately 70% in 2015 compared to the index year 2009 has been attained, while investigating how to reduce this number further in the next few years [15,16]. This reduction had no impact on the health and welfare of farm animals [17]. However, a decrease in the prevalence of AMR bacteria and/or genes in human medicine, as a consequence of the reduction in antimicrobials use (AMU) on farms, has not been evident by now, and no environmental impact as a result of this reduction has been measured [18,19]. Nevertheless, the management of AMR at the human–animal–environment interface requires the urgent implementation of the One Health approach for effective control and prevention [20]. 

**Figure 1:**
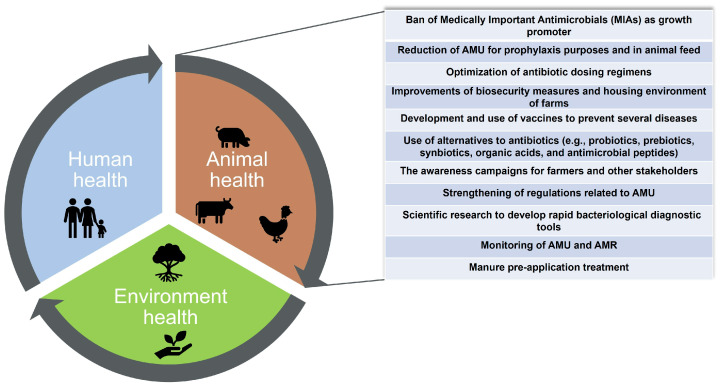
Actions conducted in livestock to tackle antimicrobial resistance from a One Health perspective. AMR, antimicrobial resistance; AMU, antimicrobial use.

The One Health approach has been recently defined by the Food and Agriculture Organization of the United Nations (FAO), the World Organization for Animal Health (WOAH, founded as OIE), the World Health Organization (WHO), and the United Nations Environment Programme (UNEP) (tripartite and UNEP) as “an integrated, unifying approach that aims to sustainably balance and optimize the health of people, animals, and ecosystems” [21]. It is noteworthy that from a One Health perspective, several actions have been carried out with respect to livestock, especially over the past decade, to limit the spread of AMR bacteria and to preserve the effectiveness of antimicrobials (Figure 1) [15,22,23,24,25,26]. However, the global effect of these actions, regarding the reduction of AMR at the human-animal-environment interface, is still under investigation, and very few scientific studies have shown encouraging results with some antimicrobials such as colistin [13,14]. Hence, the overarching goal of the present review is to provide an overview of AMU in food-producing animals and to document the current state of the art regarding the role of farm animals in the spread of AMR in humans. The impact of farm animals on the dissemination of AMR in the environment, from a One Health perspective, has been recently reviewed extensively elsewhere [27,28,29], and thus this topic is not covered in the present review. 

## 2. Antimicrobials Use (AMU) in Food-Producing Animals

**Figure 2:**
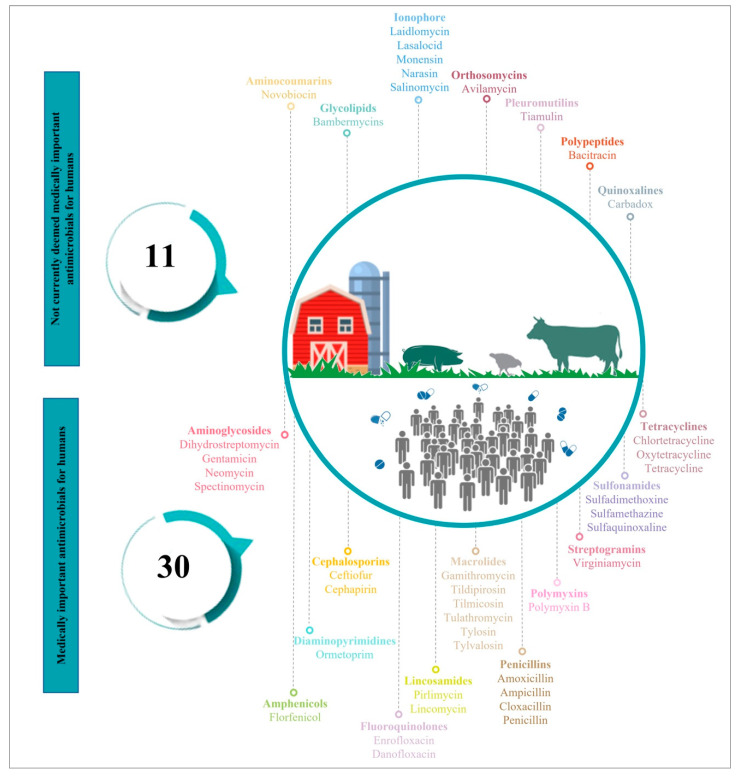
Classification of antimicrobials used in food-producing animals according to their importance for humans (Food and Drug Administration (FDA). According to the FDA, among the 41 antimicrobials (including ionophores) that are approved for use in farm animals in 2020, 30 are categorized as being medically important for humans [32]).

In farm animals, antimicrobials are used therapeutically (to treat clinically sick animals) for prophylaxis (to healthy animals at risk of infection), for metaphylaxis (to treat diseased animals in the same group as healthy animals) [20,30], and in some countries, several antimicrobials are still used in farm animals for growth promoting purposes (as a feed additive) [31]. As an example, according to the Food and Drug Administration (FDA), of the 41 antimicrobials (including ionophores) that are approved for use in farm animals in 2020 in the USA, 30 are categorized as being medically important for human medicine (Figure 2) [32]. 

Drug classification is an important tool for addressing AMR against medically important antimicrobials (MIAs). Restricting the use of these antimicrobials in both animals and humans may prolong their usefulness in the two sectors [33]. The OIE has developed a classification scheme of antimicrobials of importance to animal health (Table 1) [34]. The development of this OIE list is based on the scientific opinion of experts and is regularly updated when new information becomes available [35]. Meanwhile, the WHO has developed a scheme to classify antimicrobials with respect to their importance to human medicine into three categories: critically important, highly important, and important [36]. There is substantial overlap between the WHO and OIE list regarding the importance of some antimicrobial classes for both human and veterinary medicine such as third- and fourth-generation cephalosporins and fluoroquinolones [37]. Ultimately, both the WHO and OIE lists contribute to the development and update of national treatment guidelines and advices on prevention and risk prioritization to preserve the efficiency of antimicrobials that are essential to safeguard human and animal health [35]. 

The global trends in AMU in farm animals has been extensively discussed elsewhere [38,39,40,41,42]. A brief overview is provided here with a focus on the main findings. In 2010, the global usage of antimicrobials in farm animals was estimated at 63,151 tonnes, while this quantity was estimated at 93,309 tonnes in 2017 and projected to increase by 11.5%, ultimately reaching 104,079 tonnes by 2030 [38,39,42]. However, the aquaculture sector, despite its rapid growth globally, was not included in this estimation due to the uncertainty regarding the level of antimicrobials used in this animal production. This aspect could lead to an underestimation of the real quantities of antimicrobials used in the veterinary sector on a global scale. Interestingly, pig production had the largest projected increase in AMU globally and contributed by 45% to the total increase between 2017 and 2030 [38]. Moreover, it has also been estimated that there will be an increase of 15% for AMU in humans between 2015 and 2030 [38]. Interestingly, it was reported that about 75% of the administered antimicrobials are not absorbed in the digestive tract of farm animals and are excreted via the feces or urine, which can directly contaminate the surrounding farm environment as well as the agricultural lands and runoff following manure application as fertilizer [43]. It is noteworthy that China was the largest consumer of veterinary antimicrobials, accounting for 45% of global use in 2017, and it is expected to keep its first position as the biggest consumer in 2030 (43%) [38]. In 2018 alone, 29,774 tonnes of antimicrobials were used in farm animals in China, and 53.2% of this quantity being used as growth promoters (feed additives) [44]. In the United States, antimicrobial use in food animals was estimated to account for ~80% of the nation’s annual antimicrobial consumption (AMC) in 2010 [39]. In Canada, the Public Health Agency of Canada estimates that, in 2018, the livestock sector accounted for 78% of the total AMU, while the human and crop sectors was responsible for 21% and 1% of AMU, respectively [45]. However, the population correction unit (PCU), also referred to as a measure of biomass, that enables standardization of antimicrobial product weight (mg) per unit of animal or human biomass (kg), should be considered when estimating the AMU or AMC [45,46]. Thereby, when the animal PCU was applied in the Canadian context, animal-intended antimicrobial distribution was only 1.3 to 1.4 times that prescribed for humans [46]. Indeed, in 2018, there were roughly 21 animals for every human in Canada (an underestimate, as the population of fish being exposed to antimicrobials was not considered in these estimations) [45]. In 2017, 4122 and 6558 tonnes of active antimicrobial substances were sold for consumption in humans and for farm animals, respectively, in the 29 European Union (EU)/European Economic Area (EEA) countries [47]. According to the second ECDC/EFSA/EMA joint report (2017) on the integrated analysis of AMC and AMR in 28 countries of the EU, the average of the total consumption of antimicrobials expressed in milligrams of active substance per kilogram of estimated biomass was 124 mg/kg in humans and 152 mg/kg in farm animals [48]. Interestingly, in this report, the AMC was lower or much lower in food-producing animals than in humans in 18 of 28 countries included in the analysis. In two countries, AMC was similar, and in the eight remaining countries, AMC was higher or much higher in food-producing animals than in humans [48]. The number of countries in which the population biomass-corrected consumption was lower or much lower in food-producing animals than in humans increased to 20 in the third ECDC/EFSA/EMA joint report (2018) [47]. Ultimately, accurate global quantitative data on the use of antimicrobials in farm animals are still unknown [30], but it is estimated that the amount of antimicrobial drugs consumed by farm animals was slightly higher than the antimicrobial drugs used for humans (133 mg/kg and 118 mg/kg, respectively), leading probably to a higher selection for resistant bacteria and/or their resistance determinants in animals [49]. 

Monitoring and surveillance of AMU in animals is generally not harmonized among the different countries. As a consequence, the results are not always directly comparable [50]. Indeed, some countries used veterinary data (e.g., invoices, prescriptions), or antimicrobial sales data at the level of pharmaceutical companies, while others used data on AMU collected directly from farms [51,52]. To overcome this situation, the OIE has developed standards for monitoring the quantities of antimicrobials used in farm animals and in the aquatic sector [40]. These standards include recommendations regarding the sources of AMU data (e.g., pharmaceutical manufacturers, importers, veterinarians, farmers), the types of data that should be monitored (e.g., weight of active ingredient, dosage regimens, numbers of farm animals by species), and options for reporting AMU data (e.g., total usage by antimicrobial class, by animal species, and by route of administration) [50]. For instance, measuring the antibiotics in weight of active ingredient is inherently prone to some misinterpretations when comparing different sectors, as the dosages for one treatment and thus also the selective effect for AMR are not equal. Despite these standards, there remain a lot of discrepancies between the various quantification approaches, applied to report AMU in livestock, throughout the world [53]. Moreover, most of the data on antimicrobials intended for use in farm animals come from high income countries, creating a bias for more intensively reared animals [42]. Nevertheless, more and more countries report AMU data to the OIE, reaching in 2020 approximately 86% of the countries [42]. While considerable advancement has been made over the past decade on AMU data collection from low-income and middle-income countries (LMICs), more work needs to be carried out [2]. Further progress will depend on the possibilities of the competent authorities of these countries and on the support that international organizations (e.g., OIE, World Bank) could provide them, particularly in terms of digitization (information technology tools) and the necessary funding to address the resources (e.g., dedicated staff) needed to engage in AMU data collection [42].

The estimated predictions on AMU for 2030 or 2050 [38] should be analyzed with caution. Indeed, several countries have recently introduced restrictions on the use of antimicrobials in farm animals (e.g., ban of antimicrobials as growth promoters, prohibiting all forms of systematic AMU in farming (including prophylactic treatments), strengthening of regulations related to the sale and prescription of antimicrobials in farm animals, awareness-raising campaigns) (Figure 1). These factors should be considered when interpreting this projection. For example, in April 2017 China banned the use of colistin in feed as a growth promoter for farm animals [54,55]. This decision was possibly associated with the withdrawal of more than 8000 tonnes of colistin from the Chinese animal production sector [54]. Moreover, antimicrobial sales for farm animals across the entire EU have dropped by 43.2% on a biomass-adjusted (mg/PCU) basis from 2011 to 2020 across the 25 countries that had reported yearly data to the European Medicines Agency (EMA) [56,57]. Furthermore, in the province of Québec, Canada, a new policy restricting the use of antimicrobials of very high importance for human medicine in food-producing animals has been in force since February 2019 [58]. This category of antimicrobials is referred to in Canada as ‘Category 1’ antimicrobials. It involves, among others, third-and fourth-generation cephalosporins, polymyxins, and fluoroquinolones used in the animal production sector. This new policy prohibits the use of these antimicrobials for disease prevention in food-producing animals and indicates that veterinarians must prove that an antimicrobial of lesser importance for humans would not be effective (via antimicrobial susceptibility testing) or available before prescribing them. An important decrease in Category 1 AMU in dairy farms was observed following the implementation of this policy [59]. In addition, other factors such as the uncertainty surrounding the impact of the current COVID-19 pandemic on AMU in animal production [20], the global geopolitical changes aimed at promoting the resilience of food production systems in each country, the change in consumption habits and in the global demand for animal protein, regulatory changes (e.g., requiring fees paid by veterinary drug users) [49], the creation of national reduction targets, and the changes in data-collection systems (as well as the possibility of developing new effective alternatives to antibiotics in farm animals) could also influence the future quantities of antimicrobials that will be used in animal production.

## 3. The Link between the Use of Antimicrobials in Farm Animals and the Spread of AMR in Both Animals and Humans

### 3.1. Relationship between AMU and AMR in Farm Animals 

Several studies explored the selective effect of the level of AMU and the selection for AMR bacteria in farm animals. However, their results were not conclusive in most cases [60]. Indeed, no studies could demonstrate a significant selective effect on the intestinal population of *E. coli* following the use of colistin in pigs, whether used at therapeutic or at higher doses [61,62,63]. This was also not found for cephalosporin resistance mediated by extended-spectrum beta-lactamase (ESBL)-producing (ESBLs) *E. coli* strains of dairy herd origin [60,64]. 

On the other hand, several other studies could establish a link between AMU and AMR in farm animals [47,65,66]. In Japan, the voluntary withdrawal of the off-label use of ceftiofur in hatcheries in March 2012 was also followed by a significant decrease in broad-spectrum cephalosporin resistance in *E. coli* from healthy broilers [66]. In a pan-European analysis (data from 31 European countries) from 2014 to 2018, a significant association was observed between the consumption of third and fourth generation cephalosporins and the prevalence of ESBL and/or AmpC-producing *E. coli* in food-producing animals (broilers, turkeys, pigs and veal calves), but also for other antimicrobials such as fluoroquinolones, aminopenicillin, polymyxin, and tetracycline and different bacterial species (including *Campylobacter jejuni*) [47]. Two years after regulation was introduced to limit the use of Category 1 antimicrobials in the province of Québec, Canada (February 2019), in food-producing animals, the proportion of multidrug resistance *E. coli* isolates had significantly decreased in the sampled dairy farms (fecal and manure pit samples) [67]. In China, after the ban on the use of colistin as growth promoter (April 2017), the prevalence of the *mcr-1* gene, in pigs and poultry, significantly decreased [68,69]. Also in Europe, a decrease in AMR to the growth promoting antibiotics was seen after the ban of these drugs in farm animals [70]. However, this was different according to the animal species and the antimicrobial as well as the country. In general, this depended on the location of the different genes encoding resistance against these antimicrobials [71,72,73]. A meta-analysis on globally published data demonstrated that interventions on farms that restrict antimicrobials as feed additives appear to be most effective at reducing AMR in livestock, while narrowly targeted interventions restricting the use of single antimicrobial or a single class of antimicrobials are less likely to be effective in decreasing AMR in farm animals [74]. Also, it was not possible to conclude whether reduced use of antibiotic as feed additive alone was more effective in reducing AMR in farm animals compared with restrictions involving prophylactic indications [74]. However, in specific experimental and epidemiological studies, the selection for AMR of growth promotors has been demonstrated [71,75]. Also, at the molecular level, using multilevel mixed-effects models and a semiquantitative approach, it was shown that the restriction of AMU in farm animals was associated with a lower presence of antimicrobial resistance genes (ARGs) in bacteria isolated from animals (*vanA*, *mecA*, *bla*_CTX-M_, *mcr-1*, *aadA2*, *tet*(E), *tet*(P), *vat*(E), *sul2*, *dfrA5* and *dfrA13*) and humans (*vanA*), while no effects were detected for β-lactamases other than *bla*_CTX-M_ and the remaining *tet* genes [76].

As can be seen from the information above, the causal link between the use of antimicrobials in animal production and the selection of resistant bacteria and their genetic determinants is not universal. This indicates a complex causality involving the interaction of several other factors that determine the magnitude of selection pressure, the spread, and the persistence of AMR bacteria and genes within the animal intestinal microbiota and in the farms environment [77]. Several factors can be implicated, including the way antimicrobials are used on the farm (therapeutically, prophylactically or as a growth promoter), routes of administration, duration of antimicrobial use, veterinary control, herd size, and the level of biosecurity and sanitation as well as the genetic linkages of genes in the bacteria [74,78]. Generally, reducing the use of MIAs in livestock is associated with a reduced selective pressure of these antimicrobials on animal gut microbiota, leading to gradual reduction of the resistant bacterial population [13,79]. However, the co-existence on a single plasmid within the same bacterial strain of serval AMR genes causing co-resistance selection may always trouble the relationships [80,81]. Nevertheless, through the use of neural networks and other statistical analysis, complex relationships could be demonstrated [82,83]. This illustrates the importance of implementing a comprehensive strategy, at the farm level, that includes the reduction of all antimicrobials [84]. From an animal health and welfare perspective, a complete ban on all AMU is not advisable, and it has been shown that this is not required as long as the judicious use of these drugs is respected [74]. 

### 3.2. Relationship between AMU/AMR in Farm Animals and AMR in Humans

The link between AMR in humans and AMR in animals, and thus ultimately the use of antimicrobials in livestock and pets, is still puzzling the scientific community. Only a few studies have focused on this problem with a ‘One Health’ approach. When studying the transfer of resistance, a first distinction should be made between zoonotic bacteria and other bacteria. Since zoonotic bacteria are present in animals, and frequently without any harm, AMR is selected in animals before it infects humans. The odds that this bacterium goes back to the animal from an infected human is very unlikely. It is thus clear that resistance in zoonotic bacteria is mainly selected in animals and that there is a direct transfer of these bacteria with their AMR genes from animals to humans. The indirect transfer, however, is more complex. Hereby, AMR genes from bacteria of animals transfer to bacteria from humans. It is problematic that AMR genes are present in both animal and human bacteria as well as in bacteria in the environment, and that the contribution of resistance to any of them is thereby very hard to evaluate (as there is no difference between AMR genes). Thus, a direct source attribution of AMR genes is not possible. 

#### 3.2.1. Direct Transfer of AMR through Zoonotic Bacteria

Several studies reported that after fluoroquinolones were introduced for farm animals use in 1995, a significant increase in the prevalence of resistance toward this antimicrobial in animal and human *Campylobacter* clinical isolates was observed in several countries (e.g., USA, Spain) [80,81]. The same trend was recently confirmed in the third joint inter-agency report of the ECDC/EFSA/EMA, showing a significant positive association between fluoroquinolone resistance in *C. jejuni* from broilers and fluoroquinolone resistance of *C. jejuni* from humans [42]. This confirms the selection of the resistance in animals and the subsequent zoonotic transfer and the direct link between AMU in livestock and AMR in humans. 

However, there are differences in the methods of transmission of zoonotic bacteria that should be taken into account. This is exemplified by studies carried out on livestock-associated methicillin-resistant *Staphylococcus aureus* (LA-MRSA). Multiple sources of evidence support the role of swine as reservoirs of LA-MRSA for humans [85,86]. This microorganism has gained particular interest, from a human health perspective, since the first findings of LA-MRSA of clonal complex CC398/sequence type (ST)398 in pigs in 2005 in France [87]. This strain was originally of human origin, and a host specificity shift has occurred, after which the methicillin resistance was acquired in animals [88]. Humans (e.g., farmers, veterinarians, slaughterhouse employees) in close contact with farm animals have a greater risk of being colonised by LA-MRSA than the rest of the population [86], and a quantitative association between AMU (e.g., cephalosporins, tetracyclines, and penicillins) and MRSA in pigs and humans living and/or working on pig farms was found. This suggests that a reduction in AMU is potentially effective in decreasing MRSA carriage in pigs [89]. However, this reduction of LA-MRSA in animals does not necessarily reduce the presence of LA-MRSA in humans in the general population (which has little to no contact with farm animals). Only reducing the contact between animals and humans, such as through reducing the number of working hours in swine farms, has been demonstrated to be a stronger determinant for decreasing the dissemination of LA-MRSA to humans than AMU in livestock [76]. Moreover, it was demonstrated that multidrug-resistant *Salmonella* Typhimurium DT104 and its resistance genes were widely maintained in cattle and humans separately, with limited transmission in both direction [90]. This difference between food borne and contact borne transmission causes the differences seen between AMU in livestock and AMR in humans and ultimately the transfer of the resistant bacteria, as exemplified by *Campylobacter* and *Salmonella* (which are foodborne) and LA-MRSA (which is rather transmitted through direct contact). This causes only a temporary colonisation, rather than contamination, and as such gives different results.

#### 3.2.2. Indirect Transfer of AMR through Commensal Bacteria

The first studies investigating this transfer used in vivo models and were limited in their set up. One such study examined the transfer of vancomycin-resistant enterococci (VRE) [91], wherein six volunteers ingested a VRE isolate of chicken origin with the outcome then assessed. It was observed that there was only a temporary presence of VRE in the human intestine and that after some time, no VRE could be detected anymore [91]. However, this presence could lead to the transfer of the vancomycin resistance to human bacteria, which has been shown subsequently [91]. 

Other studies examined the potential transfer of ESBLs from animal to human bacteria. The first ex vivo studies conducted have shown that the genes were readily transferred even without selective pressure [92]. However, this may not completely reflect the more complex in vivo situation. Using robust statistical methods, the extent ot which there was a transfer was further assessed. Other studies indicated a high rate of transfer (although these data were then countered, and less transfer was assumed) [93]. 

A study conducted in the Netherlands demonstrated that 19% of community-acquired ESBL-producing *E. coli* and plasmid-mediated AmpC producing *E. coli* might possibly be attributable to foodstuffs of animal origin [94]. Meanwhile, human to human transmission in the community accounted for about 60%, and transmission between humans belonging to risk groups represented another 7% [94]. In the same country (the Netherlands), it was recently demonstrated that using a multidirectional dynamic risk model to describe the spread of ESBL-producing *E. coli* between broiler flocks, broiler farmers, and the open community, the colonization of the open community could primarily be attributed to the open community itself (62%), followed by vegetable consumption (29.5%) and contact with farmers (8.5%) [95]. Likewise, in the UK, most human bacteremia with ESBL-producing *E. coli*, involve community-associated infection of genitourinary or gastrointestinal origins, while non-human reservoirs made little contribution to invasive human disease in this country [96]. In a systematic review, it was also shown that only a proportion of human extraintestinal expanded-spectrum cephalosporin-resistant *E. coli* (ESCR-EC) infections originate from farm animals and that poultry appears to be the more likely source than other farm animals [97]. However, the specific parameters surrounding this transmission, including the magnitude and geographical extent of this issue, have yet to be understood [97]. Whole genome sequencing (WGS) analysis, using phylogenetic core genome comparisons, confirmed that *E. coli* isolates (including ESBL-producing isolates) from cross sectional surveys of livestock, retail meat, and human patients in East England were genetically distinct, suggesting that *E. coli* causing serious human invasive disease had not directly originated from livestock [98]. Similarly, comparing the genomes of ESBL-producing *E. coli* isolates from humans or human-polluted environments (e.g., human wastewater) with isolates from livestock or animal-polluted environments (e.g., animal wastewater) on Reunion Island [99] confirmed that isolates from these two reservoirs were genetically different, suggesting that livestock have very little contribution (<5%) on the acquisition of ESBL-producing *E. coli* by humans [99]. This study confirmed again the primarily role of human-to-human transmission of AMR bacteria and/or genes. Several other studies could not demonstrate the transfer [100] or, if they did so, could only demonstrate the transfer to an extremely limited extend [100,101]. Thus, these studies indicate a very limited exchange, if at all, between humans and animals. 

Nevertheless there are other studies showing a closer link between ESBL positive *E. coli* from animals (especially poultry and human strains causing infection, most likely through the food chain) [102]. On the island of Zanzibar, it was shown that the gut microbiota of healthy local people is very often colonized with the same MDR Enterobacterales (MDR-Ent) concurrently present in poultry or contaminated chicken meat [103]. Other studies also reported a link between animals and humans of the presence of colistin resistant *E. coli* strains and the subsequent transfer in persons with direct contact [104,105]. Indeed, a distinction has to be made between persons with direct contact with animals and those who have not had such contact. 

Discrepancies between studies related to the existence or not of a link between farm animals and humans regarding the transmission of AMR could be explained by several factors. The use of a variety of protocols for the determination of the susceptibility of bacteria to antimicrobials and for the detection of ARGs are probably of minor influence in the comparison of the results between studies [76]. The variety of bacteria, resistant genes, population groups, and statistically based source attributions may be of greater importance. For example, the presence of similar AMR bacteria in persons with or without contact with livestock seems to be an important factor, and thus those populations should be assessed separately. Non-transferable resistance such as mutational resistance against fluoroquinolones are of little importance when it occurs in bacteria that only temporarily colonize humans and do not cause disease. The sampled human population should also be divided into persons with and without direct contact, as their risks may be quite different. Also, studies using source attribution, based mainly on the uneven distribution of the AMR genes in the different ecosystems, represent some degree of uncertainty in the analysis. Moreover, analysis based on the different genes present may also be skewed by the use of antimicrobials in humans selecting a one-time transferred gene, and thus can amplify the magnitude of transfers. While WGS certainly has contributed to our understanding of transfer of bacteria in general, it has been of limited use to the risk assessment as it does not allow the source attribution of the different AMR genes [97]. Other more promising methods using a computational approach that integrates machine learning, WGS, gene sharing network, and mobile genetic elements analysis were recently proposed to demisting the role of farm animals in the dissemination of AMR to humans. However these methods still require further elaboration [106]. Ultimately, regardless of the evidence or not of a causal link between animals and humans regarding the transmission of AMR, veterinary medicine has been very proactive in advocating the One Health approach for AMR control, and this will certainly help maintain animal health and lead to a longer lifespan of the current available antimicrobials (Figure 1) [8,43,84].

#### 3.2.3. Link between AMU in Farm Animals and AMR in Human Bacteria

Much research has been carried out regarding the association between AMU in livestock and AMR in humans. The effect of interventions that restrict AMU in food-producing animals on AMR in humans is still under investigation [18,76]. Nevertheless, for zoonotic pathogens the effects are clear (this has been discussed in the section on direct transfer of resistance). 

After the ban of avoparcin as a growth promotor in 1995 in Denmark a decrease in the occurrence of glycopeptide-resistant *E. faecium* (GRE) in broilers, from 72.7% in 1995 to 5.8% in 2000 was measured [70,74]. The decrease in pigs was, however, much lower due to the link with a tylosin resistance gene [70]. Moreover, the rate of GRE carriers within healthy humans in Germany was assessed after the ban in 1996 and after a year the percentage carriage decreased from 13% in 1994 to 4% in 1997 [107]. It is noteworthy that the WHO has described VRE as a high-priority pathogen with urgent need of new treatments, with Enterococci containing *vanA* gene are resistant to vancomycin and teicoplanin [108]. However, it should be noted also that those strains causing infections in humans are different, and there was no effect on GRE infections in humans after the ban of avoparcin [109,110,111].

After the ban of colistin use as a growth promoter in farm animals in China, in April 2017, a significant reduction in colistin-resistant *E. coli* and the prevalence of *mcr-1* gene was observed in chicken and pigs as well as in humans in comparison with data collected before the entry into force of this policy [13]. Similarly, in 2005, when hatcheries in Quebec (Canada) voluntarily withdrew the use of ceftiofur (third generation cephalosporin) in ovo for a one year period, there was a high significant decline in ceftiofur resistant *E. coli* and *Salmonella enterica* serovar Heidelberg in both retail chicken and humans isolates [112]. Interestingly, there was also a significant positive association between consumption of third- and fourth-generation cephalosporins and fluoroquinolones in farm animals (broilers, turkeys, pigs and veal calves) and resistance to theses antimicrobials in invasive *E. coli* isolates from humans [47]. 

A systematic review, commissioned by the WHO, demonstrated that the reducing of AMU in livestock has been associated with a decrease in the prevalence of AMR bacteria in animals by about 15% [18]. This resulted in an even larger reduction in humans (24%), but this could only be assessed for humans having direct contact with such animals (and overall the analysis was less robust) [18]. The results of the studies that are ongoing in the Netherlands will be interesting. In that country, a reduction of about 70% of AMU compared to the index year 2009 was obtained through government control. There has already been a sharp decline in the overall resistance in *E. coli* strains from livestock [113]. Whether this will result in a decrease in resistance in human pathogens is still under investigation. Care should be taken, as AMU in humans affect the resistance prevalence in those bacteria and, as such, will involve a challenging analysis. 

## 4. Conclusions

Antimicrobial resistance compromises the possibility of effectively treating bacterial diseases in humans and animals and represents a global challenge to agriculture and public health. The extensive use of antimicrobials in animals and humans is being recognized as a major driver for the emergence and spread of AMR worldwide. The high occurrence of resistant bacteria and their resistance determinants in the intestinal microbiota of farm animals is a major concern for animal and human health (as well as for the environment). 

The amount of antimicrobial drugs (biomass-adjusted (mg/PCU)) consumed by farm animals, based on the available global data, was only slightly higher than the antimicrobial drugs used for humans. Banning the global use of growth promotors will likely bring the use of antimicrobials in livestock lower than in humans. Care should be taken with the interpretation of these data, as the use of mg is not very precise as some antibiotics are more potent than others. 

The contribution of farm animals to the spread of AMR to humans continues to be debated within the scientific community, with a lot of uncertainties and contradicting results having emerged. Therefore, there is definitely a need for more research in this field. Nevertheless, it can be concluded that AMR in zoonotic bacteria is of animal origin and that these resistant bacteria can cause infections in humans and thus pose a threat to human health as possible resistant bacterial infections. The contribution of commensal resistant bacteria is still a matter of debate, although we can conclude that the impact is probably much smaller than first anticipated. 

Very specific interventions that restrict the use of only a single antimicrobial or antimicrobial class in farm animals was associated with a limited effectiveness in reducing AMR in both sectors. It is believed that part of this can be attributed to co-resistance selection. Overall reduction of AMU may have much larger effects. This has been shown in animal bacteria, although the impact on human bacteria is still a matter of debate. 

Nevertheless, combating AMR (as well as preserving the effectiveness of antimicrobials in both human and veterinary medicine) will have major benefits, and there is a need for accelerating the implementation of the One Health approach which needs strong multisectoral collaboration and partnerships between all stakeholders including physicians, veterinarians, environmental professionals, researchers, pharmaceutical companies, hospitals, policymakers, and patients for reducing AMU and AMR. Finally, while global public health efforts continue to focus on the management of the COVID-19 pandemic, this public health priority should not overshadow the efforts required to contain the current “not so silent” AMR pandemic. 

## Figures and Tables

**Table 1:** OIE classification of antimicrobials regarding their importance for animal health.

Category	Antimicrobial Classes
Veterinary Critically Important Antimicrobial Agents (VCIA)	Aminoglycosides Amphenicols Cephalosporins (third and fourth generation) Diaminopyrimidines MacrolidesPenicillins Fluoroquinolones (Quinolones second generation) Sulfonamides Tetracyclines
Veterinary Highly Important Antimicrobial Agents (VHIA)	Ansamycin–Rifamycins Cephalosporins first and second generations Ionophores Lincosamides Phosphonic acid Pleuromutilins Polypeptides (e.g., colistin) Quinolones first generation
Veterinary Important Antimicrobial Agents (VIA)	Aminocoumarin Arsenical Bicyclomycin Fusidic Acid Orthosomycins Quinoxalines Streptogramins Thiostrepton

## Data Availability

Not applicable.
